# MAFLD associated with COPD via systemic inflammation independent of aging and smoking in men

**DOI:** 10.1186/s13098-022-00887-w

**Published:** 2022-08-16

**Authors:** Tsubasa Tsutsumi, Dan Nakano, Machiko Kawaguchi, Ryuki Hashida, Shinobu Yoshinaga, Hirokazu Takahashi, Keizo Anzai, Takumi Kawaguchi

**Affiliations:** 1 Division of Gastroenterology, Department of Medicine, Kurume University School of Medicine, Kurume, Japan; 2 Department of Orthopedics, Kurume University School of Medicine, Kurume, Japan; 3 Medical Examination Section, Medical Examination Part Facilities, Public Utility Foundation Saga Prefectural Health Promotion Foundation, Saga, Japan; 4 Division of Metabolism and Endocrinology, Faculty of Medicine, Saga University, Saga, Japan

**Keywords:** Metabolic associated fatty liver disease, Chronic obstructive pulmonary disease, Systemic inflammation, CRP/albumin ratio, Steatosis

## Abstract

**Background and aim:**

Metabolic dysfunction and associated systemic inflammation are risk factors for chronic obstructive pulmonary disease (COPD) and COPD is highly prevalent in men. We investigated the impact of metabolic-associated fatty liver disease (MAFLD) and MAFLD-related systemic inflammation on COPD in men.

**Methods:**

We enrolled 2,041 men with fatty liver. Patients were classified into the COPD (n = 420/2041) and non-COPD (n = 1621/2041) groups. COPD and its high-risk group were diagnosed using the Japanese Respiratory Society Disease statement. Systemic inflammation was evaluated using the C-reactive protein (CRP)/albumin ratio. Independent factors for COPD were investigated by multivariate analysis and decision-tree analysis.

**Results:**

The prevalence of MAFLD was significantly higher in the COPD group than in the non-COPD group. In multivariable analysis, in addition to heavy smoking and aging, MAFLD was identified as an independent factor for COPD (OR 1.46, 95% CI 1.020–2.101, P = 0.0385). Decision-tree analysis showed that MAFLD, rather than heavy smoking, was the most influential classifier for COPD in non-elderly men (14% in MAFLD vs 6% in non-MAFLD groups). MAFLD was also the second most influential factor in elderly men who were not heavy smokers. In both groups, the CRP/albumin ratio was the first classifier for COPD (16% in the high CRP/albumin ratio group vs 3% in the low CRP/albumin ratio group of non-elderly men).

**Conclusions:**

MAFLD is an independent predictor of COPD in men. MAFLD had a significant impact on COPD through systemic inflammation in men of all ages who were not heavy smokers. MAFLD may be useful to broadly identify COPD in men.

## Introduction

In patients with fatty liver disease, the prevalence of extra-hepatic diseases is high, emphasizing the systemic involvement of metabolic dysfunctions [1]. Non-alcoholic fatty liver disease (NAFLD) has been used as a concept of fatty liver, however, NAFLD excludes other chronic diseases and moderate amounts of alcohol consumption. More importantly, NAFLD does not require the presence of metabolic dysfunction, resulting in the metabolic heterogeneity of NAFLD. This metabolic heterogeneity is thought to cause a mixture of patients at low and high risk of extra-hepatic disease [2, 3].

Recently, an international expert panel proposed a new concept for fatty liver disease: metabolic (dysfunction) associated with fatty liver disease (MAFLD) [2]. Since the presence of metabolic dysregulation is mandatory for the diagnosis of MAFLD, MAFLD captures patients with a high risk for extrahepatic complications, including chronic kidney disease and colorectal adenomas [4]. In addition, we previously reported that MAFLD identifies patients at a high risk of atherosclerotic cardiovascular disease [5]. Moreover, the changing NAFLD to MAFLD had been supported by various associations including the European Liver Patient's Association [6].

The prevalence of chronic obstructive pulmonary disease (COPD) is high in patients with NAFLD [7]. NAFLD is also associated with the severity of COPD, and the risk of COPD is particularly high in adult men [8]. Smoking and aging are well-established risk factors for COPD [9]. However, one-fourth of adults with COPD have never smoked, indicating the presence of other risk factors [10]. Metabolic syndrome is also common in patients with COPD, with a prevalence ranging from 23 to 53% [11, 12]. A PRISMA-compliant meta-analysis demonstrated that metabolic syndrome was significantly associated with a 1.53-fold increased risk of COPD [13]. Through systemic inflammation, metabolic syndrome is associated with various conditions, including interleukin-6 levels [14], which is also a characteristic of COPD [15].

Various parameters are associated with systemic inflammation. C-reactive protein (CRP) is a popular inflammatory molecule related to the exacerbation of COPD [16]. Similarly, serum albumin concentrations and negative acute phase response protein levels are lower in patients with COPD [17]. The CRP/albumin ratio has been widely used as a biomarker of systemic inflammation [18]. The CRP/albumin ratio is strongly associated with more severe metabolic dysfunction in premenopausal women [19]. The CRP/albumin ratio is also higher in patients with type 2 diabetes and diabetic nephropathy [20]. Recently, the CRP/albumin ratio has been reported as a novel biomarker to predict rehospitalization and frequent exacerbations in patients with acute COPD exacerbations [21].

This study aimed to investigate the impact of MAFLD on COPD in men. We also investigated the impact of metabolic dysfunction and systemic inflammation on COPD in patients with MAFLD.

## Patients and methods

### Study design and ethics

This was a single-center observational cohort study in Japan. The protocol conformed to the ethical guidelines of the 1975 Declaration of Helsinki, as reflected by the prior approval from the institutional review board of Kurume University School of Medicine (ID 20,050). An opt-out approach was used to obtain informed consent from the patients, and personal information was protected during data collection.

### Study population and selection of patients for analysis

**Fig. 1:**
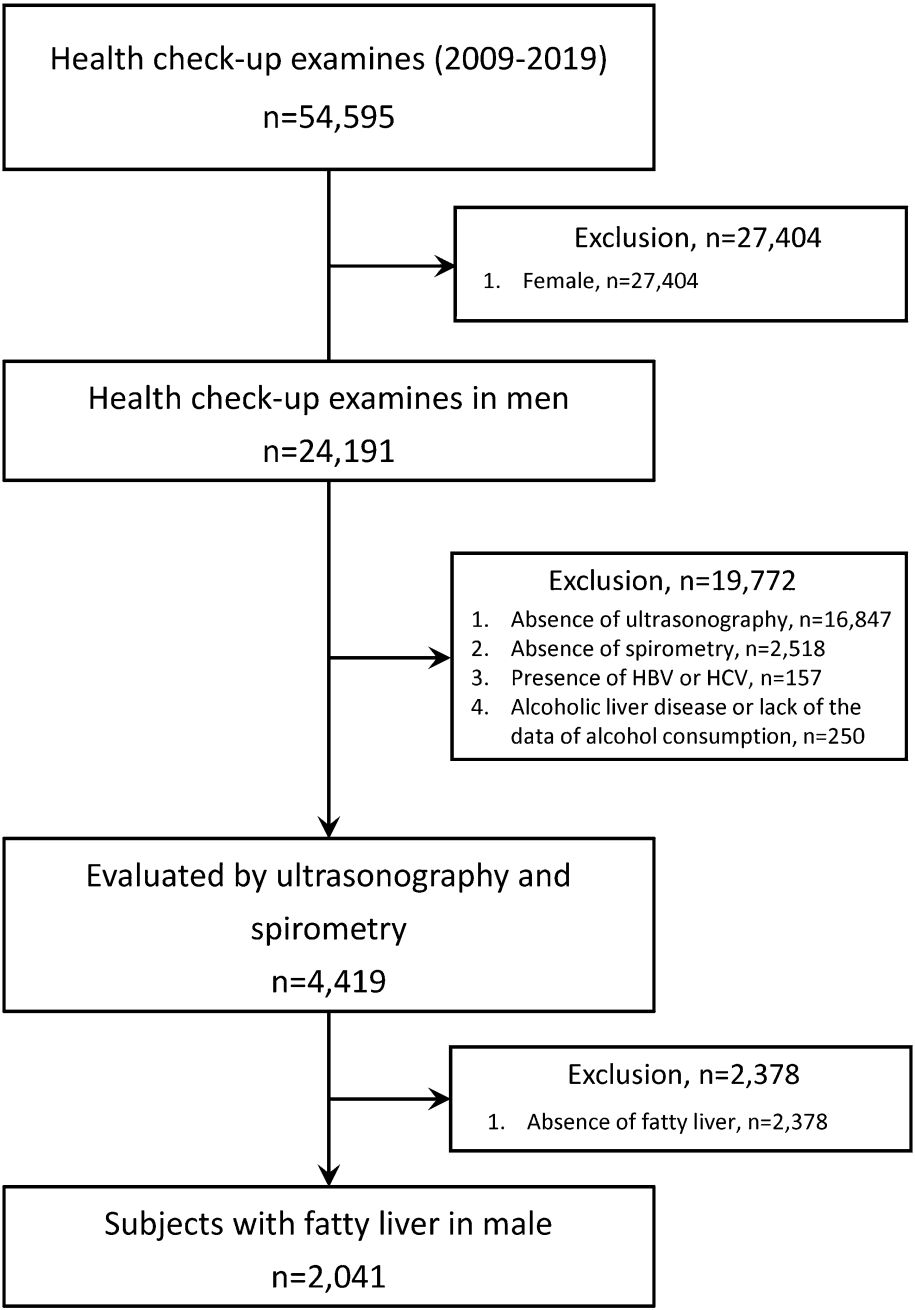
Flow chart of patient selection

We enrolled 54,595 Asian participants who underwent health check-up examinations at the Saga Health and Clinical Examination Centre in Japan from January 2009 to March 2019 (Fig. 1). We excluded 27,404 women, and we excluded 19,772 of the 24,191 men for the following reasons: absence of ultrasonography, absence of spirometry test, presence of hepatitis B surface antigen, presence of anti-hepatitis C virus antibody, presence of alcoholic liver disease (pure ethanol ≥ 60 gms/day), and a lack of alcohol consumption data. From the remaining 4419 participants, we excluded 2378 participants because of the absence of fatty liver. Finally, we examined 2,041 men with fatty liver disease (Fig. 1).

### Data collection

All data were collected prospectively at the time of the medical check-up [5]. The following information was obtained using a self-reported questionnaire: age, sex, current smoking habits, alcohol consumption, comorbidities, and medication use. In the clinical review, we obtained the following data: body mass index (BMI), visceral adiposity (waist circumference ≥ 90 cm), blood pressure, presence/absence of diabetes, hypertension, and dyslipidemia, which were diagnosed according to standard criteria [22–24].

### Biochemical analysis

Blood samples were obtained after an overnight fast, and the following biochemical parameters were measured: complete blood cell count, aspartate aminotransferase (AST), alanine aminotransferase (ALT), alkaline phosphatase, γ-glutamyl transpeptidase (GGT), lactate dehydrogenase, total protein, albumin, total bilirubin, total cholesterol, triglycerides (TG), high-density lipoprotein cholesterol (HDL), low-density lipoprotein (LDL) cholesterol, amylase, blood urea nitrogen, creatinine, estimated glomerular filtration rate, CRP, uric acid, electrolytes, fasting glucose, hemoglobin A1c (HbA1c), and cholinesterase.

### Calculation of FIB-4 index

The FIB-4 index was calculated using age, serum levels of AST and ALT, and platelet count [25].

### CRP/albumin ratio

The serum levels of CRP and albumin were measured simultaneously during the health check examination. The CRP/albumin ratio was calculated by dividing the serum CRP level by the serum albumin level [26].

### Diagnosis of fatty liver

Fatty liver was diagnosed by abdominal ultrasonography as previously described [27]. All tests were performed by medical sonographers certified by the Japan Society of Ultrasonics in Medicine.

### Diagnosis of MAFLD

MAFLD was diagnosed according to the criteria [2]. Briefly, the criteria include evidence of fatty liver, in addition to one of the following: overweight/obesity, presence of type 2 diabetes mellitus, or lean/normal weight with evidence of metabolic dysfunction. Overweight/obesity was defined as BMI ≥ 23 kg/m^2^, and type 2 diabetes mellitus was defined as HbA1c ≥ 6.5% or specific drug treatment. Metabolic dysfunction was defined as the presence of at least two metabolic risk abnormalities: 1) waist circumference ≥ 90 cm, 2) systolic blood pressure ≥ 130 mmHg, diastolic blood pressure ≥ 85 mmHg, or specific drug treatment; 3) plasma TG ≥ 150 mg/dL or specific drug treatment; 4) plasma HDL cholesterol < 40 mg/dL or specific drug treatment; and 5) prediabetes (fasting glucose levels 100–125 mg/dL or HbA1c 5.7%–6.4%) [2]. Homeostasis model assessment-insulin resistance score and plasma high-sensitivity C-reactive protein level are metabolic risk abnormalities [2]; however, these were not available in our dataset.

### Definition of heavy and non-heavy smoking

We used pack years to evaluate the amount of cigarette smoking. It is calculated by multiplying the number of packs of cigarettes smoked per day by the number of years the person has smoked. We classified individuals into the following two groups according to their smoking habits: 1) heavy smoking; pack-years ≥ 60; 2) non-heavy smoking; 0 ≤ pack-years < 60, as previously described [28].

### Spirometry measurement

Lung function was evaluated by spirometry with an electronic diagnostic spirometer (SP-770 COPD, FUKUDA DENSHI CO., LTD., Japan) for all participants, according to the American Thoracic Society quality criteria [29]. The results were expressed as a percentage of the predictive values based on age, height, and sex. All tests were performed during the early hours of the day. Three to six trials were performed for each subject. All tests were performed by a clinical laboratory technician.

### Diagnosis of COPD

COPD is a chronic irreversible disease [30]; therefore, it is important to detect COPD in the high-risk group as recommended by the Japanese Respiratory Society [31]. Therefore, we integrated COPD with its high-risk group as a COPD group.

Airway obstruction was evaluated by the result of spirometry. COPD was defined as the forced expiratory volume % in one second (FEV1%) less than 70% according to the COPD management guidelines [32].

COPD high-risk group was defined by the percentage of predicted forced expiratory volume in 1 s (%FEV1) which was calculated by the FEV1 to predictive FEV adjusted by age, sex, and height [33]. COPD's high-risk group was defined by a %FEV1 less than 80% [31].

### Statistical analysis

Continuous variables are expressed as medians, ranges, or numbers. Categorical variables are expressed as frequencies and percentages. Differences between the two groups were analyzed using the Mann–Whitney *U* test. Logistic regression analysis was used to identify independent factors associated with the presence of COPD. Since metabolic syndrome, visceral obesity, and diabetes are inclusion criteria for MAFLD, these metabolic factors were not used as explanatory variables in multivariable analysis because they are co-founding factors. Explanatory variables were selected in a stepwise manner, minimizing the Bayesian information criterion [34].

Data are expressed as odds ratios (ORs) and 95% confidence intervals (CIs). A decision-tree algorithm was constructed to show profiles associated with the presence of COPD [35]. All *P*-values were two-tailed, and a value < 0.05 was considered statistically significant. Multivariate stepwise analysis and decision tree analysis were performed using JMP Pro16 (SAS Institute, Cary, NC, USA).

## Results

### Differences in patient characteristics between the COPD and non-COPD groups

The participant characteristics are summarised in Table 1. The COPD group comprised 20.6% of enrolled patients. Age was significantly higher in the COPD group than in the non-COPD group. The prevalence of smoking habits and pack years was significantly higher in the COPD group than in the non-COPD group. (Table 1).

**Table 1:** The difference in patients’ characteristics between the COPD and non-COPD groups

	COPD group		Non-COPD group		*P*
	Median (IQR)	Range (min–max)	Median (IQR)	Range (min–max)	
Number	20.6% (420/2041)	N/A	79.4% (1621/2041)	N/A	N/A
Age	58 (50–64.8)	27–84	51 (43–60)	21–85	< 0.0001
Presence or past smoking habit (Yes/No)	86.0%/14.0% (361/59)	N/A	71.9%/28.1% (1166/455)	N/A	< 0.0001
Pack years (SUM of past and present)	30 (0–40)	0–160	14 (0–28)	0–111	< 0.0001
Pack years ≥ 60 (Yes/No)	10.5%/89.5% (44/376)	N/A	3.5%/96.5% (57/1564)	N/A	< 0.0001
Alcohol intake habit (None/Yes)	32.9%/67.1% (138/282)	N/A	31.2%/68.8% (1115/506)	N/A	0.5188
Dairy alcohol consumption (none/1–19 g/20–39 g/40–59 g)	32.9%/16.2%/36.0%/15.0% (138/68/151/63)	N/A	31.3%/23.1%/31.2%/14.4% (507/375/506/233)	N/A	0.0178
MAFLD/non-MAFLD	90.5%/9.5% (380/40)	N/A	85.9%/14.1% (1393/228)	N/A	0.0148
Body mass index (kg/m^2^)	25.2 (23.2–27.7)	15.7–43.2	24.9 (23.1–26.9)	15.6–40.6	0.1282
Waist circumference (cm)	91 (86.5–98)	70–137.5	89 (84.5–95)	67–123.5	< 0.0001
Visceral adiposity (Presence/Absence)	57.9%/42.1% (243/177)	N/A	46.9%/53.1% (760/861)	N/A	< 0.0001
Systolic blood pressure (mmHg)	124 (114–132)	84–197	120 (112–130)	80–190	0.0006
Diastolic blood pressure (mmHg)	80 (70–86)	50–118	78 (70–84)	48–134	0.2075
Type 2 diabetes mellitus (Presence/Absence)	11.4%/88.6% (48/372)	N/A	8.3%/91.7% (135/1486)	N/A	0.0475
Hypertension (Presence/Absence)	46.2%/53.8% (194/226)	N/A	38.8%/61.2% (629/992)	N/A	0.0063
Hypertriglyceridemia (Presence/Absence)	48.1/51.9% (202/218)	N/A	40.8%/59.2% (661/960)	N/A	0.0068
Depressed HDL-cholesterol (Presence/Absence)	16.2%/83.8% (68/352)	N/A	11.2%/88.8% (181/1440)	N/A	0.0050
%VC	96.0 (86.8–110.4)	50.1–149.2	108.4 (100–117.2)	77.3–157.7	< 0.0001
FEV1%	69.5 (65.7–75.3)	40.3–90.9	79.7 (76.5–83.1)	70–98.1	< 0.0001
%FEV1	75.9 (70.4–79.9)	43.2–91.5	96.1 (89.2–103.6)	80.0–110.8	< 0.0001
Red blood cell count (× 10^4^/µL)	487 (464–514)	384–610	495 (469–520)	258–657	0.0019
Hemoglobin (g/dL)	15.2 (14.5–15.9)	12.7–19.1	15.3 (14.6–15.9)	8.1–18.7	0.3179
Hematocrit (%)	44.5 (42.5–46.6)	36.8–54.2	44.7 (42.9–46.4)	28.5–56.2	0.4685
White blood cell count (/µL)	5700 (5100–7375)	2700–15,100	5700 (4900–6800)	2200–15,400	0.0446
Platelet count (× 10^4^/µL)	22.7 (19.5–26.5)	7.3–75.9	23.4 (20.3–26.7)	6.4–51.5	0.0437
AST (U/L)	23 (19–29)	10–90	23 (19–25)	6–164	0.0928
ALT (U/L)	28 (20–38)	6–277	28 (20–40)	6–290	0.8053
Lactate dehydrogenase (U/L)	168 (153–188)	70–311	164 (149–182)	56–431	0.0081
ALP (U/L)	213 (178–256)	73–502	209 (179–249)	73–1409	0.2035
GGT (U/L)	43 (30–71)	12–381	38((26–61)	8–609	0.0002
Total protein (g/dL)	7.2 (7.0–7.4)	6.2–8.4	7.2 (7.0–7.4)	5.7–8.5	0.5728
Cholinesterase (U/L)	367 (322–404)	188–532	369 (331–408)	166–641	0.0883
Albumin (g/dL)	4.4 (4.3–4.6)	3.1–5.1	4.5 (4.3–4.6)	3.2–5.3	< 0.0001
Total bilirubin (mg/dL)	0.7 (0.6–0.9)	0.2–2.2	0.8 (0.6–1.0)	0.2–3.3	0.0630
Total cholesterol (mg/dL)	202(183–223)	122–331	207(186–229)	120–357	0.0107
HDL-cholesterol (mg/dL)	48 (41–60)	24–116	52 (45–60)	26–164	< 0.0001
LDL-cholesterol (mg/dL)	124 (107–141)	39–260	129 (111–150)	54–262	0.0011
Triglycerides (mg/dL)	136 (98–206)	31–990	123 (90–178)	33–1474	0.0005
Fasting glucose (mg/dL)	103 (96–113)	81–220	99 (94–109)	77–330	< 0.0001
HbA1c (%)	5.8 (5.5–6.1)	4.3–11.1	5.7 (5.5–6.0)	4.4–12.8	< 0.0001
CRP (mg/dL)	0.1 (0.1–0.17)	0.01–3.99	0.1 (0.08–0.12)	0.01–3.52	< 0.0001
BUN (mg/dL)	13.7 (11.6–15.9)	6.6–37.5	13.6 (11.7–15.8)	6.4–30.3	0.8189
Creatinine (mg/dL)	0.8 (0.7–0.9)	0.43–1.64	0.8 (0.74–0.9)	0.48–2.4	0.0193
eGFR (mL/min/1.73 m^2^)	78.4 (69.7–89.8)	34.9–149.4	79.8 (70.4–89.1)	25.3–142.6	0.1243
Uric acid (mg/dL)	6.4 (5.6–7.1)	3.4–11.9	6.3 (5.4–7.1)	0.7–10.8	0.3569
Sodium (mmol/L)	141 (140–143)	136–145	141 (140–142	134–147	0.6563
Potassium (mmol/L)	4.2 (4.0–4.4)	3.3–5.3	4.2 (4.0–4.3)	3.3–5.3	0.3399
Chloride (mmol/L)	105 (104–107)	99–111	105 (104–107)	98–110	0.1171
CRP/albumin ratio	0.023 (0.021–0.038)	0.002–1.05	0.022 (0.018–0.027)	0.002–0.892	< 0.001
FIB-4 index	1.135 (0.847–1.484)	0.225–6.985	0.937 (0.702–1.300)	0.281–9.404	< 0.001

*COPD* chronic obstructive pulmonary disease, *MAFLD* metabolic associated fatty liver disease, *VC* vital capacity, *HDL-cholesterol* high-density lipoprotein-cholesterol, *FEV* forced expiratory volume, *FVC* forced vital capacity, *AST* aspartate aminotransferase, *ALT* alanine aminotransferase, *GGT* γ-glutamyl transpeptidase, *LDL-cholesterol* low-density lipoprotein-cholesterol, *HbA1c* hemoglobin A1c, *CRP* C-reactive protein, *BUN* blood urea nitrogen, *GFR* glomerular filtration rate, *FIB-4* fibrosis-4

The prevalence of MAFLD was greater than 90% in the COPD group and significantly higher than that in the non-COPD group. The serum albumin level was significantly lower in the COPD group than in the non-COPD group. Furthermore, the CPR/albumin ratio was significantly higher in the COPD group than in the non-COPD group. The FIB-4 index was significantly higher in the COPD group than in the non-COPD group (Table 1).

### Multivariate analyses of independent factors for the COPD

**Fig. 2:**
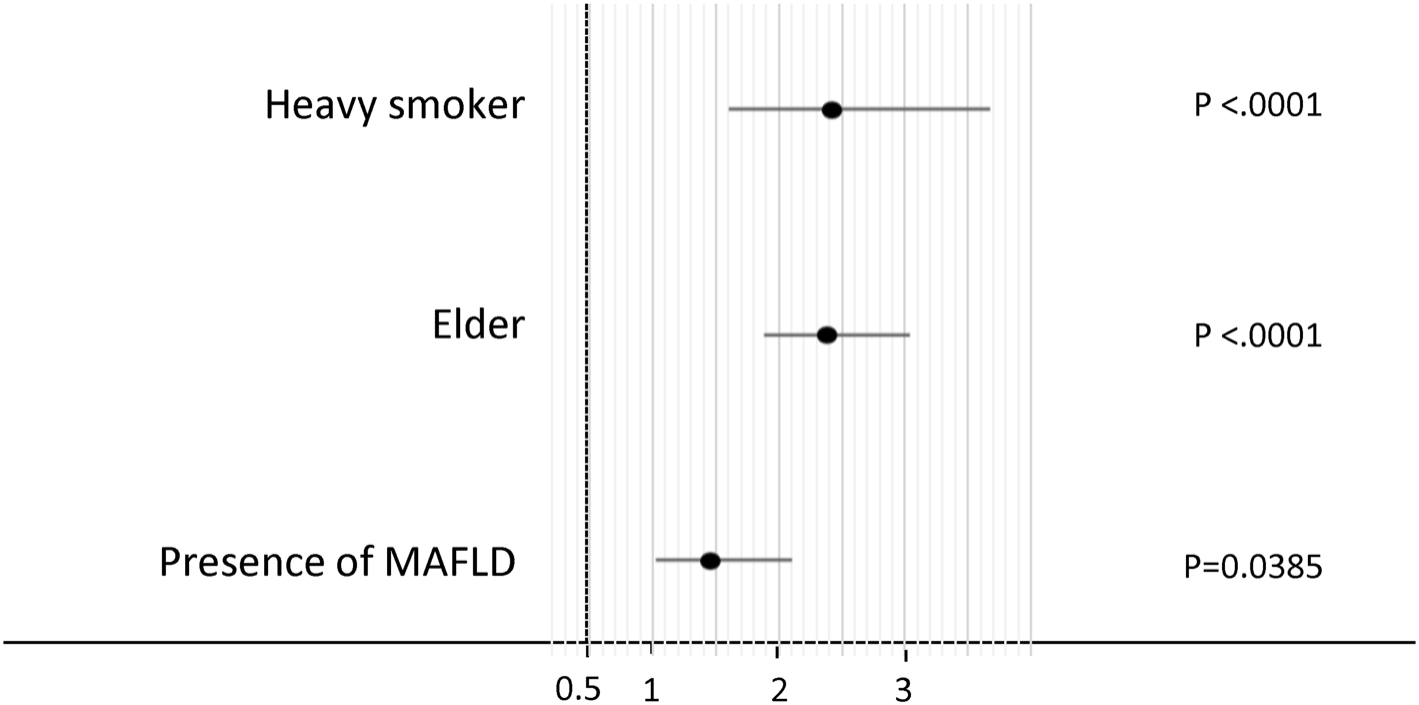
Independent factors for the presence of chronic obstructive pulmonary disease in all subjects. *MAFLD* metabolic-associated fatty liver disease

In the stepwise selection procedure, heavy smoking, aging, and MAFLD were selected for logistic regression analysis. Age was categorized by the cut-off value of 50 years old as previously described [36]. In multivariate analysis, the independent factors for COPD were MAFLD (OR 1.46, 95% CI 1.020–2.101, P = 0.0385), heavy smoking (OR 2.43, 95% CI 1.599–3.581, P < 0.0001), and aging (OR 2.39, 95% CI 1.878–3.037, P < 0.0001) (Fig. 2).

### Impact of COPD according to decision tree analysis

**Fig. 3:**
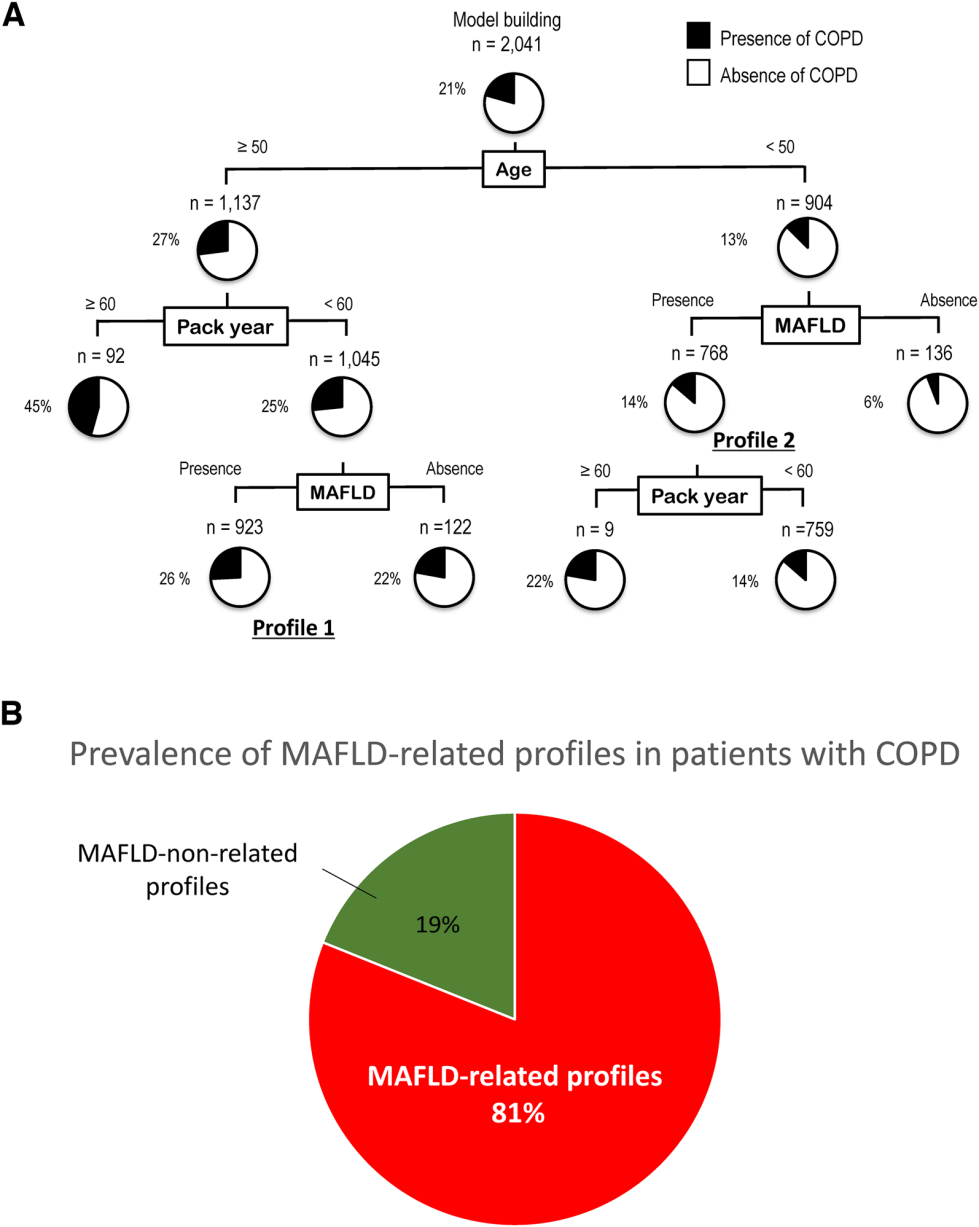
**A** Profiles associated with chronic obstructive pulmonary disease were evaluated by decision tree analysis (**B**) Prevalence of metabolic-associated fatty liver disease-related profiles in patients with chronic obstructive pulmonary disease. *COPD* chronic obstructive pulmonary disease, *MAFLD* metabolic-associated fatty liver disease

We investigated the profiles of the presence of COPD using decision tree analysis (Fig. 3). The initial classifier is aging. Of the subjects aged ≥ 50 years, 27% had COPD. The second and third classifiers were smoking and MAFLD, respectively. In the subjects with < 60 pack-years and MAFLD, 26% had COPD (Profile 1). Of the subjects aged < 50 years, 13% had COPD. Of these, the second classifier was MAFLD rather than smoking (Profile 2). The prevalence of COPD was 14% in subjects aged < 50 years who had MAFLD. On the other hand, the prevalence was 6% in subjects aged < 50 years with non-MAFLD.

Thus, decision tree analysis demonstrated two MAFLD-related profiles. The prevalence of patients with MAFLD-related COPD profiles in all patients with COPD was calculated by the following formula. A) All patients with COPD: n = 420 (Table 1), B) MAFLD-related COPD: 923 by 0.26 (Profile 1) and 768 by 0.14 (Profile 2) = 347.5 Thus, subjects with these MAFLD-related COPD profiles were calculated as B divided by A (347.5/420), resulting in 81.1%. (Fig. 3).

### Characteristics of patients with MAFLD-related profiles

As a sub-analysis, we investigated the characteristics of patients with COPD in subjects with two MAFLD-related profiles: Profile 1, ≥ 50 years old, less than 60 pack-years, and MAFLD) (Table 2), and Profile 2, < 50 years old and MAFLD (Table 3).

**Table 2:** The patients’ characteristics in subjects with MAFLD-related Profiles 1

	Profile 1 ≥ 50 years old, less than 60 pack-years, and MAFLD	
	Median (IQR)	Range (min–max)
Number	45.2% (923/2041)	N/A
Age	60 (55–65)	50–85
Body mass index (kg/m^2^)	25.1 (23.6–26.8)	15.7–36.8
Waist circumference (cm)	90 (86.5–95)	69.5–123
Visceral adiposity (Presence/Absence)	53.6%/46.4% (495/428)	N/A
Systolic blood pressure (mmHg)	126 (118–134)	84–197
Diastolic blood pressure (mmHg)	80 (72–86)	50–118
Type 2 diabetes mellitus (Presence/Absence)	12.2%/87.8% (113/810)	N/A
Hypertension (Presence/Absence)	52.0%/48.9% (471/452)	N/A
Hypertriglyceridemia (Presence/Absence)	45.0%/55.0% (416/507)	N/A
Depressed HDL-cholesterol (Presence/Absence)	12.2%/87.8% (113/810)	N/A
Alcohol intake habit (None/Yes)	30.0%/70.0% (277/646)	N/A
Dairy alcohol consumption (None/1–19 g/20–39 g/40–59 g)	30.0%/23.4%/32.9%/13.7% (277/216/304/126)	N/A
Presence or past smoking habit (Yes/No)	76.5%/23.5% (706/217)	N/A
Pack years (sum of past and present)	20 (0.8–34.5)	0–58.8
Pack year ≥ 60 (Yes/No)	10.5%/89.5% (44/376)	N/A
Pulmonary functions		
%VC	105.4 (96.3–114.9)	50.1–151.4
FEV1%	76.9 (72.3–80.5)	48.4–90.5
%FEV1	92.7 (83.2–101.6)	43.6–151.2
Prevalence of COPD (Presence/Absence)	25.8%/74.2% (238/685)	N/A
Biochemical examinations		
Red blood cell count (× 10^4^/µL)	487 (461–509)	370–603
Hemoglobin (g/dL)	15.1 (14.4–15.8)	8.9–19.1
Hematocrit (%)	44.3 (42.3–46.2)	30.1–75.9
White blood cell count (/µL)	5500 (4800–6700)	2,200–15,400
Platelet count (× 104/µL)	22.2 (18.9–25.2)	8.8–75.9
AST (U/L)	23 (19–28)	12–154
ALT (U/L)	26 (19–35)	6–290
Lactate dehydrogenase (U/L)	167 (151–187)	56–330
ALP (U/L)	207 (174–250)	73–1409
GGT (U/L)	38 (27–62)	12–609
Total protein (g/dL)	7.2 (6.9–7.4)	6.0–8.4
Cholinesterase (U/L)	360 (322–396)	180–641
Albumin (g/dL)	4.4 (4.3–4.6)	3.4–5.1
Total bilirubin (mg/dL)	0.8 (0.6–1.0)	0.2–3.0
Total cholesterol (mg/dL)	205(186–227)	122–313
HDL-cholesterol (mg/dL)	51 (44–60)	26–123
LDL-cholesterol (mg/dL)	126 (109–147)	55–225
Triglycerides (mg/dL)	130 (95–181)	33–1474
Fasting glucose (mg/dL)	103 (97–115)	78–330
HbA1c (%)	5.9 (5.6–6.2)	4.3–12.8
CRP (mg/dL)	0.1 (0.09–0.13)	0.01–3.99
BUN (mg/dL)	14.1 (12.2–16.3)	8.0–37.5
Creatinine (mg/dL)	0.8 (0.74–0.9)	0.43–1.7
eGFR (mL/min/1.73 m^2^)	75.1 (66.6–83.3)	31.6–149.4
Uric acid (mg/dL)	6.3 (5.4–7.0)	0.7–11.0
Sodium (mmol/L)	141 (140–143)	134–146
Potassium (mmol/L)	4.2 (4.0–4.4)	3.3–5.3
Chloride (mmol/L)	105 (104–107)	98–111
CRP/albumin ratio	0.227 (0.02–0.429)	0.002–1.05
FIB-4 index	1.267 (0.966–1.628)	0.225–6.985

*COPD* chronic obstructive pulmonary disease, *MAFLD* metabolic associated fatty liver disease, *VC* vital capacity, *HDL-cholesterol* high-density lipoprotein-cholesterol, *FEV* forced expiratory volume, *FVC* forced vital capacity, *AST* aspartate aminotransferase, *ALT* alanine aminotransferase, *GGT* γ-glutamyl transpeptidase, *LDL-cholesterol* low-density lipoprotein-cholesterol, *HbA1c* hemoglobin A1c, *CRP* C-reactive protein, *BUN* blood urea nitrogen, *GFR* glomerular filtration rate, *FIB-4* fibrosis-4

**Table 3:** The patients’ characteristics in subjects with MAFLD-related Profiles 2

	Profile 2 < 50 years old and MAFLD	
	Median (IQR)	Range (min–max)
Number	37.6% (768/2041)	N/A
Age	43 (39–47)	21–49
Body mass index (kg/m^2^)	25.9 (24.4–28.2)	18.4–137.5
Waist circumference (cm)	91.5 (87–97.5)	71–123.5
Visceral adiposity (Presence/Absence)	59.0%/41.0% (453/315)	N/A
Systolic blood pressure (mmHg)	120 (110–128)	89–190
Diastolic blood pressure (mmHg)	78 (70–84)	48–134
Type 2 diabetes mellitus (Presence/Absence)	7.9%/92.1% (61/707)	N/A
Hypertension (Presence/Absence)	36.1%/63.9% (277/491)	N/A
Hypertriglyceridemia (Presence/Absence)	48.9%/51.1% (375/393)	N/A
Depressed HDL-cholesterol (Presence/Absence)	15.8%/84.2% (121/647)	N/A
Alcohol intake habit (None/Yes)	33.4%/66.6% (257/511)	N/A
Dairy alcohol consumption (None/1–19 g/20–39 g/40–59 g)	33.5%/19.9%/29.8%/16.8% (257/153/229/129)	N/A
Presence or past smoking habit (Yes/No)	70.2%/29.8% (539/229)	N/A
Pack years (Sum of past and present)	10 (0–22.5)	0–100
Pack year ≥ 60 (Yes/No)	3.5%/96.5% (57/1564)	N/A
Pulmonary functions		
%VC	107.5 (98.0–117.2)	70.6–155.5
FEV1%	80.7 (77.0–84.0)	54.4–98.1
%FEV1	93.2 (85.6–101.2)	48.6–134.4
Prevalence of COPD (Presence/Absence)	13.6%/86.4% (105/663)	N/A
Biochemical examinations		
Red blood cell count (× 10^4^/µL)	508 (483–531)	396–657
Hemoglobin (g/dL)	15.5 (14.8–16.1)	12.0–18.7
Hematocrit (%)	45.2 (43.5–46.9)	36.4–56.2
White blood cell count (/µL)	6,050 (5100–7000)	3000–13,600
Platelet count (× 104/µL)	24.4 (21.5–27.6)	7.3–51.5
AST (U/L)	24 (19–30)	6–164
ALT (U/L)	34 (24–51)	8–277
Lactate dehydrogenase (U/L)	165 (151–182)	84–431
ALP (U/L)	209 (182–248)	73–506
GGT (U/L)	43 (29–68)	10–449
Total protein (g/dL)	7.2 (7.0–7.4)	5.9–8.4
Cholinesterase (U/L)	384 (346–427)	208–613
Albumin (g/dL)	4.6 (4.4–4.7)	3.1–5.2
Total bilirubin (mg/dL)	0.7 (0.6–0.9)	0.2–3.0
Total cholesterol (mg/dL)	207 (186–229)	120–357
HDL-cholesterol (mg/dL)	49 (43–57)	24–164
LDL-cholesterol (mg/dL)	131 (114–152)	54–262
Triglycerides (mg/dL)	136 (93–201)	31–1233
Fasting glucose (mg/dL)	98 (93–104)	77–312
HbA1c (%)	5.6 (5.4–5.8)	4.5–12.6
CRP (mg/dL)	0.1 (0.08–0.15)	0.01–3.39
BUN (mg/dL)	12.8 (11.0–14.9)	6.5–26.3
Creatinine (mg/dL)	0.8 (0.73–0.9)	0.48–2.4
eGFR (mL/min/1.73 m^2^)	85.1 (76.3–94.1)	25.3–142.6
Uric acid (mg/dL)	6.5 (5.6–7.3)	0.8–11.9
Sodium (mmol/L)	141 (140–142)	136–146
Potassium (mmol/L)	4.2 (4.0–4.3)	3.3–5.3
Chloride (mmol/L)	105 (104–107)	98–110
CRP/Albumin ratio	0.0222 (0.020–0.032)	0.002–0.892
FIB-4 index	0.725 (0.578–0.911)	0.281–5.214

*COPD* chronic obstructive pulmonary disease, *MAFLD* metabolic associated fatty liver disease, *VC* vital capacity, *HDL-cholesterol* high-density lipoprotein-cholesterol, *FEV* forced expiratory volume, *FVC* forced vital capacity, *AST* aspartate aminotransferase, *ALT* alanine aminotransferase, *GGT* γ-glutamyl transpeptidase, *LDL-cholesterol* low-density lipoprotein-cholesterol, *HbA1c* hemoglobin A1c, *CRP* C-reactive protein, *BUN* blood urea nitrogen, *GFR* glomerular filtration rate, *FIB-4* fibrosis-4

Subjects with MAFLD-related Profile 1 accounted for 45.2% of all subjects; their median age was 60 years, and their median BMI was 25.1. Their smoking habit was 76.5%, the median pack years was 20, and the prevalence of COPD was 25.8%. In addition, the prevalence of visceral adiposity, hypertension, diabetes, and hypertriglyceridemia was 59.0%, 36.1%, 7.9%, and 48.9%, respectively. The median FIB-4 index and CRP/albumin ratio were 1.267 and 0.227, respectively.

Subjects with MALFD-related Profile 2 accounted for 37.6% of all subjects, the median age was 43 years, and the median BMI was 25.1 (Table 3). The prevalence of visceral adiposity, hypertension, diabetes, and hypertriglyceridemia was 53.6%, 52.0%, 12.2%, and 45.0%, respectively. The median FIB-4 index and CRP/albumin ratio were 0.725 and 0.0222, respectively.

The reference values for CRP/albumin ratio are 0.002–0.0025 based on the reference values of CRP and albumin of < 0.01 mg/dL and 4–5 g/dL, respectively. Therefore, the CRP/albumin ratio in Profiles 1 and 2 were higher than the reference value.

### Decision-tree analysis of factors associated with COPD in subjects with MAFLD-related profiles

**Fig. 4:**
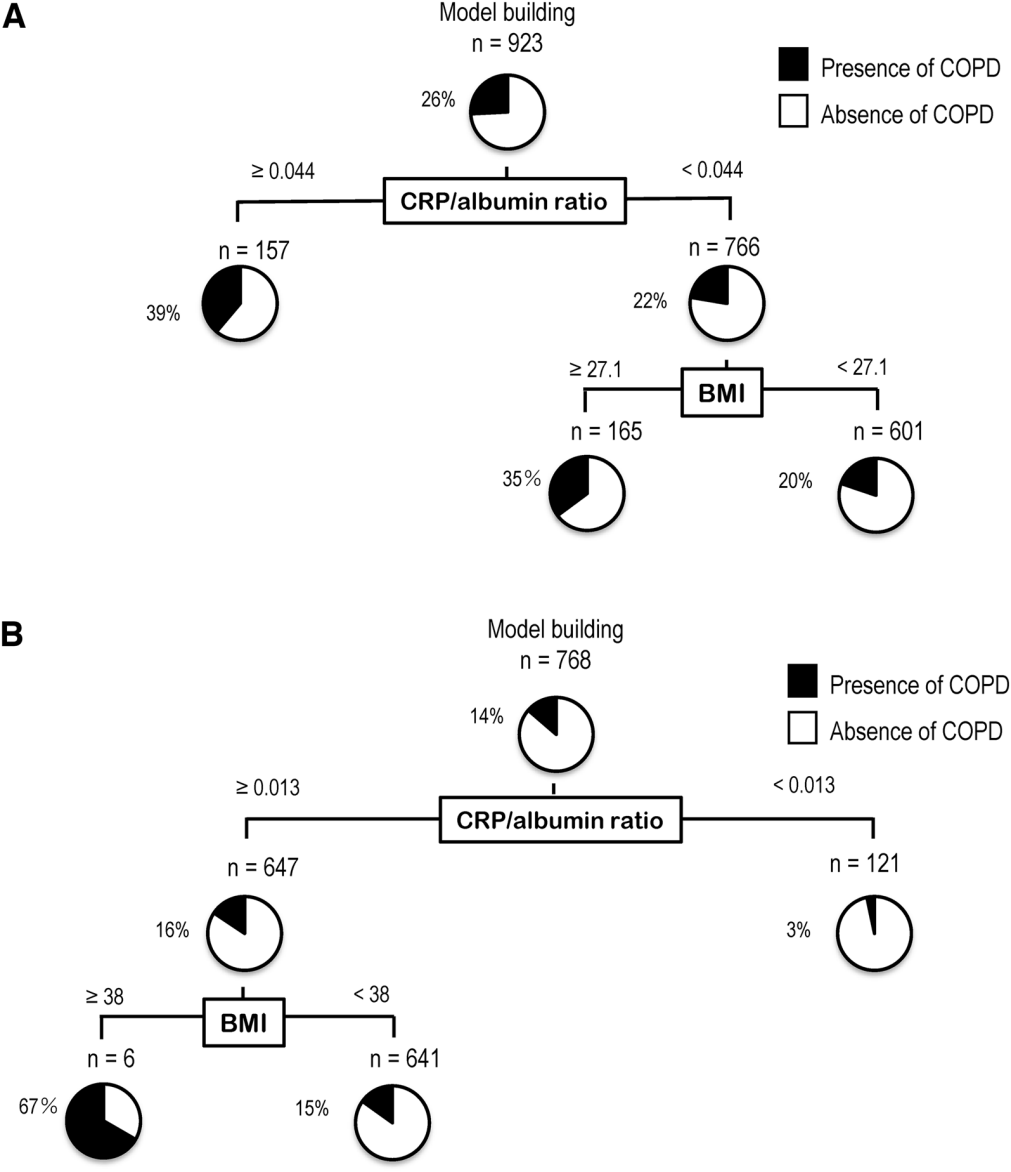
**A** Decision tree analysis for factors associated with COPD in patients with metabolic-associated fatty liver disease-related Profile 1. **B** Decision tree analysis for factors associated with chronic obstructive pulmonary disease in patients with metabolic-associated fatty liver disease-related Profile 2. *COPD* chronic obstructive pulmonary disease, *CRP* C-reactive protein, *BMI* body mass index

We investigated factors associated with COPD in subjects with MAFLD-related Profile 1 using the following explanatory variables: BMI, HbA1c, FIB-4 index, and CRP/albumin ratio. In the decision tree analysis, the initial classifier was CRP/albumin ratio. Among the subjects with a CRP/albumin ratio ≥ 0.044, 39% had COPD (Fig. 4A). On the other hand, in the subjects with a CRP/albumin ratio < 0.044, 22% had COPD.

In subjects with MAFLD-related Profile 2, the initial factor for COPD was the CRP/albumin ratio (Fig. 4B). Of the subjects with a CRP/albumin ratio ≥ 0.013, 16% had COPD. In contrast, in subjects with a CRP/albumin ratio < 0.013, 3% had COPD.

## Discussion

In this study, we first investigated whether MAFLD was an independent factor for the presence of COPD in men. In particular, MAFLD had a significant association with subjects with the following profiles:1) ≥ 50 years old with less than 60 pack-years of smoking and 2) < 50 years old, which accounted for 89% of patients with COPD. Decision-tree analysis revealed that CRP/albumin, an index of systemic inflammation, was the most important factor for COPD.

This cohort data, obtained from 2009 to 2019, was based on health check examinees. The prevalence of fatty liver was 46.1% (2,041/4,419) for all male subjects, which is similar to its prevalence in the general population according to Japanese guidelines [37]. We enrolled only men because the association between NAFLD and COPD is stronger in men than that in women [7]. Furthermore, the Nippon COPD Epidemiology (NICE) Study which examined the general Japanese population revealed that the prevalence of COPD in females was very low, less than 1/3 of that in men. [38] The prevalence of MAFLD was 40.1% (1,773/4,419), which is also in good agreement with previous reports (30.2% to 46.7%) [39, 40]. Moreover, the prevalence of COPD was 21% in all subjects, which is in good accordance with previous reports (14.6% to 41%) of patients with NAFLD [41, 42]. In addition, aging and long-term smoking were risk factors for COPD in previous studies [9], and our study showed the same trend that median age, smoking rate, and heavy smoking were all higher in the COPD group than in the non-COPD group. Thus, our database appears to correspond with the general characteristics of the population in Japan.

We demonstrated that the presence of MAFLD was an independent factor for the presence of COPD independent of aging and heavy smoking. In previous studies, the association between COPD and NAFLD remains controversial. Several studies reported that NAFLD is highly prevalent in patients with COPD [7, 43]. In contrast, one Japanese study reported a low prevalence of NAFLD in patients with COPD [44]. These differences may be due to the heterogeneous metabolic features in patients with NAFLD. Metabolic abnormalities, including obesity and type 2 diabetes mellitus, are reportedly associated with the development and progression of COPD [7]. Comorbidity of metabolic abnormalities is an inclusion criterion for the diagnosis of MAFLD. Thus, MAFLD may be an independent factor in COPD.

We employed decision-tree analysis to examine the profiles associated with COPD. MAFLD was identified as a classifier in subjects who were ≥ 50 years old and had a < 60 pack-year smoking history. MAFLD was also a classifier in subjects < 50 years of age, regardless of smoking status. These profiles accounted for greater than 80% of the patients with COPD. Thus, we have revealed that MAFLD is extensively associated with COPD. The reason for the extensive impact of MAFLD on COPD remains unclear. However, one would think that changes in the clinical phenotypes of COPD may be a possible reason. It is now emerging that up to 50% of patients with COPD have metabolic dysfunction as a comorbidity [45]. Furthermore, recent studies have shown a direct association between metabolic dysfunction and progressive lung pathology in patients with COPD [45, 46]. In fact, in our cohort, patients with COPD had a higher prevalence of metabolic dysfunction, including visceral adiposity, type 2 diabetes mellitus, hypertension, and dyslipidemia compared to patients without COPD. Thus, MAFLD may be extensively associated with COPD because of the emerging impact of metabolic dysfunction in patients with COPD.

We also performed a sub-analysis to investigate the factors associated with COPD in patients with MAFLD-related profiles. In this analysis, we included various metabolic abnormalities and inflammatory indexes, such as the CRP/albumin ratio. We would like to investigate the impact of IL-6 on COPD as a biomarker of systemic inflammation. However, this information was not available because all participants were health check examinees and there was no preserved serum. Thus, we employed the CRP/albumin ratio instead of the IL-6.

In both MAFLD-related Profiles 1 and 2, the CRP/albumin ratio was the initial classifier for COPD. COPD can be caused by various pathogeneses, including systemic inflammation and reactive oxygen species. CRP is positively correlated with IL-6 levels, a major inflammatory cytokine [47]. IL-6 has been reported to be inversely correlated with FEV1% and is associated with increased mortality in patients with COPD [48, 49]. In addition, serum albumin is known to scavenge reactive oxygen species by a free cysteine residue [50]. A meta-analysis showed that serum albumin levels are even lower in patients with stable COPD, suggesting the importance of a deficit in systemic inflammation in COPD [17]. Thus, CRP/albumin may be the most important factor for COPD morbidity in patients with MAFLD.

This study had some limitations. First, this was a cross-sectional study conducted in a single center in Japan. Second, the cohort comprised only Asians. Third, we could not evaluate dietary and exercise habits. Further international multicentre prospective studies with an evaluation of lifestyle habits should be conducted.

In conclusion, we showed that MAFLD was an independent factor for the presence of COPD in men. MAFLD was associated with elderly non-heavy smokers and non-elderly individuals, accounting for 80% of COPD cases. Furthermore, CRP/albumin had the greatest impact on COPD. Thus, MAFLD may be widely associated with COPD via systemic inflammation in men.

## Data Availability

The datasets used and/or analysed during the current study available from the corresponding author on reasonable request. Data sharing statement: no additional data available.

## Abbreviations

MAFLD: Metabolic associated fatty liver disease
COPD: Chronic obstructive pulmonary disease
NAFLD: Non-alcoholic fatty liver disease
IL-6: Interleukin-6
CRP: C-reactive protein
BMI: Body mass index
AST: Aspartate aminotransferase
ALT: Alanine aminotransferase
GGT: γ-Glutamyl transpeptidase
TG: Triglycerides
HDL-cholesterol: High-density lipoprotein-cholesterol
LDL-cholesterol: Low-density lipoprotein-cholesterol
HbA1c: Hemoglobin A1c
FIB-4 index: Fibrosis-4 index
FEV: Forced expiratory volume
FVC: Forced vital capacity
OR: Odds ratio
CI: Confidence interval
